# Investigation of QT Dispersion and T-Peak to T-End/Corrected QT Ratio in Multisystem Inflammatory Syndrome in Children

**DOI:** 10.7759/cureus.43086

**Published:** 2023-08-07

**Authors:** Özlem Turan, Murat Ciftel

**Affiliations:** 1 Pediatric Cardiology, Antalya Research and Training Hospital, Antalya, TUR; 2 Pediatric Cardiology, Sanliurfa Education and Research Hospital, Sanliurfa, TUR

**Keywords:** multisystem inflammatory syndrome, covid-19, te-tp, ventricular arrhythmia, qt dispersion

## Abstract

Introduction

Multisystem inflammatory syndrome in children (MIS-C) is characterized by hyperinflammation, heart involvement, and multiorgan failure, which develop following coronavirus disease 2019. Ventricular arrhythmias have been identified during this syndrome. It is known that the risk of ventricular arrhythmia is associated with ventricular repolarization changes. The aim of this study was to investigate the corrected QT interval, QT dispersion, T wave peak-to-end interval, and T-peak to T-end/corrected QT ratio in MIS-C.

Methods

The study included 35 patients diagnosed with MIS-C and 35 subjects as the control group. The ventricular diameters, ejection fraction, valve insufficiency, and coronary artery were examined in both groups using echocardiography. Corrected QT interval, QT dispersion, T wave peak-to-end interval, and T-peak to T-end/corrected QT ratio were determined by 12-lead electrocardiogram.

Results

The patient group had increased corrected QT interval (p<0.05), QT dispersion (p = 0.001), T-peak to T-end interval (p=0.001), and T-peak to T-end/corrected QT ratio (p = 0.001) compared to the control group. Moreover, there was a correlation between increased QT dispersion, T-peak to T-end, T-peak to T-end/corrected QT ratio, and decreased ejection fraction (r = -0.51 and p = 0.001, r = -0.71 and p < 0.001, r = -0.69 and p < 0.001, r = -0.56 and p < 0.001, respectively).

Conclusions

Our study demonstrated increased QTc interval, QT dispersion, T-peak to T-end interval, T-peak to T-end/corrected QT ratio in MIS-C. This result may indicate an increased risk of ventricular arrhythmia for these patients.

## Introduction

Coronavirus disease 2019 (COVID-19) is an infectious disease caused by the severe acute respiratory syndrome coronavirus 2 (SARS-CoV-2). Although children usually have a mild form of the infection, some children later develop multisystem inflammatory syndrome (MIS), with symptoms similar to those of toxic shock syndrome or Kawasaki disease. It is thought to result from postinfectious dysregulation of the immune system [1]. After cluster of cases have been identified, the U.S. Centers for Disease Control and Prevention (CDC) and the World Health Organization (WHO) proposed a case definition for MIS [2,3]. MIS in children (MIS-C) is a severe disease and can lead to death. It has signs of hyperinflammation and multiorgan failure. Children with MIS may develop myocarditis, left ventricular systolic dysfunction, and valvular insufficiency. In most cases, levels of troponin and N-terminal pro-brain natriuretic peptide (NT -proBNP), which are markers of cardiac involvement, are elevated. Due to heart failure, inotropic support and rarely extracorporeal membrane oxygenation may be required [4].

Electrocardiogram (ECG) abnormalities of varying degrees have been noted in MIS. ST-segmental changes, corrected QT (QTc) prolongation, and ventricular premature beats may also occur [5,6]. Serious ventricular arrhythmias such as ventricular tachycardia and ventricular fibrillation have been noted in these patients. Ventricular tachycardia or ventricular fibrillation can lead to significant hemodynamic collapse, resulting in high mortality [7-9]. Ventricular premature beats and QTc prolongation may indicate susceptibility to ventricular arrhythmias in these patients.

An enlarged QTc interval, QT dispersion, the interval between T-peak and T-end (Tp-e), and the ratio between T-peak and T-end/QTc (Tp-e/QTc) on the ECG are markers of susceptibility to ventricular arrhythmias and have been identified in previous studies [10-14]. QT dispersion was first introduced into clinical practice in 1990. QT dispersion was defined as the difference between the maximum QT interval and the minimum QT interval on a 12-lead ECG [10]. QT dispersion reflects the heterogeneity of ventricular repolarization, a precursor of ventricular arrhythmias [11,12]. The Tp-e interval is defined as the interval between the peak of the T wave and the end of the T wave. It is also a marker of ventricular repolarization function [13]. Recently, the Tp-e/QTc ratio, which is recognized as a new index, has been proposed as a more accurate measure of the variability of ventricular repolarization [14]. The aim of this study was to investigate the QTc interval, QT dispersion, Tp-e interval, and Tp-e/QTc ratio, and the risk of ventricular arrhythmias in MIS-C patients.

## Materials and methods

Study population

The study was designed as a prospective observational study and included patients diagnosed with MIS-C between October 2020 and March 2021. Approval for the study was obtained from the local ethics committee, and informed consent was obtained from all participants (2021-04-21T21- 46-28, Republic of Turkey, Ministry of Health, Health Services General Directorate). The diagnosis MIS-C was made according to the diagnostic criteria established by the Committee of the Centers for Disease Control and Prevention. The criteria included age < 18 years, the presence of fever, laboratory evidence of inflammation, and evidence of clinically severe disease requiring hospitalization involving multiple organs (more than two) (cardiac, renal, respiratory, hematologic, gastrointestinal, dermatologic, or neurologic organs), no alternative plausible diagnoses, positivity of current or recent infection with COVID-19 by RT-PCR, serology, or antigen testing, or exposure within four weeks before the onset of symptoms. The study included 35 MIS-C patients, who presented during the six months’ period, between 2 and 17 years of age, and 35 healthy, sex- and age-matched control subjects. The inclusion criteria for the patient group were as follows: children aged 2-17 years with a diagnosis MIS. Outpatients with myalgia or innocuous heart murmur without any history of disease were included as a control group. The exclusion criteria were as follows: obesity, smoking, hypertension, and congenital or acquired heart disease. Those whose echocardiographic and ECG measurements were insufficient to obtain data were excluded from the study.

After a general physical and cardiac examination, bedside ECG and echocardiography were performed in the patients diagnosed with MIS-C. Sedation was not required in any patient to obtain data. In the patient group, measurements were performed before initiation of inotropic therapy, intravenous immunoglobulin, systemic glucocorticoid, or anakinra. QT interval was determined by ECG, and the longest value was recorded. The Bazett formula was used to calculate the QTc interval. The QT dispersion was determined from the difference between the longest QT interval and the shortest QT interval on a 12-lead ECG. The interval between the peak of the T wave and the end point of the T wave was measured on precordial leads. Data from the largest interval were used. On the basis of these measurements, the Tp-e/QTc ratio was calculated. Systolic and diastolic ventricular wall diameters and systolic and diastolic ventricular internal diameters were measured with the M-mode echocardiogram, and the ejection fraction was determined. In addition, valvular insufficiency and coronary arterial diameters were determined. Height, weight, and systolic and diastolic blood pressures were measured in all patients. Systolic and diastolic blood pressures were measured in the supine position with a mercury manometer on the right brachial artery after resting for at least 5 minutes before examination. Body mass index (BMI) was calculated by dividing body weight in kilograms by the square of height in meters.

Echocardiographic examination

Two-dimensional M-mode echocardiography recordings (Vivid S60N, GE, Horten, Norway) were performed in all subjects. The 7-MHz transducer was used in children younger than 4 years. The 3-MHz transducer was used in children older than 4 years. During the study, a single-lead ECG was recorded continuously. Left ventricular systolic function was assessed with an M-mode echocardiogram in the parasternal long-axis view. Mitral insufficiency detected by color Doppler in the patients with MIS-C was performed on the basis of jet length measurement. It was defined as grade 1 if jet length was 1.5 cm or less, grade 2 if jet length was between 1.5 and 2.9 cm, grade 3 if jet length was between 3.0 and 4.4 cm, and grade 4 if jet length was more than 4.5 cm. Grade 1 was accepted as mild insufficiency, grade 2 as moderate insufficiency, and grades 3 and 4 as severe insufficiency [15].

ECG evaluation

**Figure 1:**
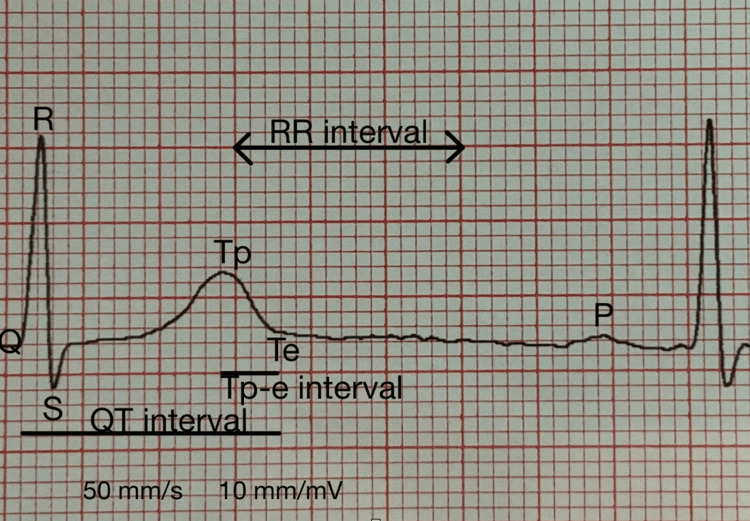
Measurement of Qtc and Tp-e intervals

Twelve-lead ECGs of the patients were recorded at rest and supine at a rate of 50 mm/s (Nihon Kohden, Tokyo, Japan). All ECGs were scanned and transferred to a PC to reduce measurement errors and then magnified by 400% using Adobe Photoshop software. Patients with sinus rhythm were included in the study. The data from subjects whose T wave end point was not clearly identified on the ECG were not recorded. The QT interval was defined as the interval between the onset of the QRS complex and the end of the T wave (Figure 1). Measurements of the QT interval were made on all leads, and the longest QT interval was recorded. The QT dispersion was determined as the difference between the maximum and minimum QT intervals (10-14). The QTc interval was calculated using the Bazett formula: QTc = QT√(R-R interval) [16]. The Tp-e interval is defined as the interval between the peak of the T wave and the end of the T wave in the ECG. For the Tp-e interval, measurements were made on the precordial leads. In these measurements, the longest Tp-e interval was recorded. Tp-e/QTc interval ratios were calculated from these measurements [10-14].

Statistical analysis

All data from the patient and control groups were written into the Excel program. Then, the data in Excel were transferred to the SPSS program, and the statistical analysis was performed. The SPSS version 15.0 statistical program (SPSS Inc., Chicago, IL, USA) was used for the statistical analysis. All values are expressed as median, mean plus or minus the standard deviation. The Pearson chi-square procedure was used for sex, and the Shapiro-Wilk test for normal distribution was performed for all other variables. The nonparametric Mann-Whitney U test was performed when variables did not conform to the normal distribution. Spearman correlation analysis was used for correlations. A p-value of less than 0.05 was considered statistically significant.

## Results

In both groups, clinical characteristics were similar in terms of age, sex, and body mass index- standard deviation scores (BMI-SDS) (Table 1). For echocardiographic M-mode findings, diastolic interventricular septal wall thickness, diastolic left ventricular posterior wall thickness, systolic interventricular septal wall thickness, and systolic left ventricular posterior wall thickness were similar between the groups. However, in the MIS-C group, left ventricular end-diastolic and left ventricular end-systolic diameters were significantly increased. In addition, the MIS-C group had decreased ejection fraction compared to the control group (p < 0.001, respectively) (Table 1).

**Table 1:** Clinical characteristics and results of conventional echocardiography in MIS-C A p-value of <0.05 was considered statistically significant MIS-C, multi-system inflammatory syndrome in children; BMI-SDS, body mass index- standard deviation scores; SBP, systolic blood pressure; DBP, diastolic blood pressure; IVSd, interventricular septal wall thickness diastolic; LVIDd, left ventricular internal dimension diastolic; LVPWd, left ventricular posterior wall thickness diastolic; IVSs, interventricular septal wall thickness systolic; LVIDs, left ventricular internal dimension systolic; LVPWs, left ventricular posterior wall thickness systolic; EF, ejection fraction

	MIS-C (n=35)	Controls (n=35)	P-value
Age (years)	8.60±3.24	8.74±2.93	0.66
Female/male	17/18	16/19	0.72
BMI-SDS	1.57±2.2	1.28±2.4	0.61
SBP (mmHg)	95.5±16.9	106.7±6.9	0.04
DBP (mmHg)	60.2±10.3	65.7±6.0	0.04
IVSd (mm)	6.20±1.13	6.08±1.24	0.68
LVIDd (mm)	43.40±5.38	40.62±5.31	0.04
LVPWd (mm)	5.77±1.28	5.68±1.40	0.79
IVSs (mm)	9.71±1.34	9.68±2.02	0.95
LVIDs (mm)	27.82±4.74	25.37±3.83	0.04
LVPWs (mm)	9.28±1.56	9.11±1.49	0.64
EF	53.7±13.5	66.2±3.5	0.001

In the patient group, the ejection fraction was less than 55% in 19 (54%) patients. An ejection fraction of less than 55% was considered impaired left ventricular systolic function. Coronary artery dilatation (Z-score between 2 and 2.5) was noted in four (11%) patients. However, there was no patient with coronary artery aneurysm. Eighteen (51%) of the patients had mitral insufficiency (severe mitral insufficiency in four patients, moderate mitral insufficiency in six patients, and mild mitral insufficiency in eight patients). In addition, minimal pericardial effusion was noted in five patients.

Comparison of the patient group with the control group showed that the patient group had prolonged QTc interval (p < 0.05), QT dispersion (p=0.001), Tp-e interval (p=0.001), and Tp-e/QTc ratio (p=0.001) (Table 2). Correlation analysis showed correlation between increased QTc interval, QT dispersion, Tp-e interval, and Tp-e/QTc ratio, and decreased ejection fraction (r = -0.51 and p = 0.001, r = -0.71 and p < 0.001, r = - 0.69 and p < 0.001, r = -0.56 and p < 0.001, respectively). There was a significant correlation between QT dispersion (r = 0.63 and p < 0.001), Tp-e interval (r = 0.54 and p < 0.001), and Tp-e/QTc ratio (r = 0.51 and p < 0.001), and severity of mitral insufficiency.

**Table 2:** Electrocardiographic measurements in MIS-C and control groups A p-value <0.05 was considered statistically significant MIS-C, multi-system inflammatory syndrome in children; QTc, corrected QT interval; Tp-e, T- peak to T-end interval; Tp-e/QTc ratio, T-peak to T-end/corrected QT ratio

ECG parameters	MIS-C (n=35)	Controls (n=35)	P-value
QTc, ms	430.71±20.79	420.57±19.80	0.04
Maximum QT, ms	345.57±22.38	339.28±26.57	0.28
Minimum QT, ms	311.28±25.47	317.42±26.38	0.32
QT dispersion, ms	34.28±14.55	21.85±10.15	0.001
Tp-e, ms	62.85±12.02	53.42±10.83	0.001
Tp-e/QTc ratio	0.14±0.02	0.12±0.02	0.001

Severe ventricular tachycardia and ventricular fibrillation were detected in one of the patients included in the study.

## Discussion

COVID-19 is a disease caused by SARS-CoV-2. Children with COVID -19 are often asymptomatic or have only mild symptoms. However, MIS-C may develop around six to eight weeks after infection with COVID-19. Symptoms of the disease are similar to those of toxic shock syndrome, Kawasaki disease, and hemophagocytic disease. Although the exact cause is unknown, it is thought to result from postinfectious dysregulation of the immune system [1]. Most children with MIS-C have either a positive nasopharyngeal RT-PCR test or an antibody test for infection [2,3]. COVID-19 infection can lead to myocardial damage. Myocarditis, hypoxic injury, cardiac microvascular injury, right heart dysfunction, stress cardiomyopathy, and systemic inflammatory response syndrome are the causes. In MIS-C, cardiac injury is likely mediated by immune activation. Hyperinflammation is the main finding of MIS.

Cardiac involvement is common and perhaps the most important and leading cause of mortality. Patients with MIS-C may develop myocarditis, left ventricular systolic dysfunction, and valvular insufficiency. Pericardial effusions and coronary artery dilatation are less common. Most patients have elevated troponin and NT-proBNP levels, which are markers of cardiac involvement [4]. In addition, ventricular tachycardia and ventricular fibrillation have been noted in MIS-C, causing severe hemodynamic collapse. These patients may require rarely extracorporeal membrane oxygenation due to hemodynamic collapse [8,9].

The QT interval includes the ventricular depolarization phase and the subsequent repolarization phase [17]. Hereditary ion channel disorders, prolongation of the QT interval due to medications, or metabolic abnormalities have been found to be associated with an increased incidence of ventricular arrhythmias [18]. QT dispersion is a measure of abnormal repolarization and may predict ventricular arrhythmias [10]. An enlarged Tp-e interval indicates the abnormal propagation of ventricular repolarization and is also associated with an increased risk of ventricular arrhythmias [10-13]. Recently, the Tp-e/QTc ratio, which is recognized as a new index, has been proposed as a more accurate measure of the variability of ventricular repolarization. The Tp-e/QTc ratio is associated with ventricular transmural dispersion during repolarization [14,19,20]. Increased QTc values, QT dispersion, Tp-e interval, and Tp-e/QTc ratio were found in the MIS-C patients enrolled in our study. In our study, we sought to investigate the risk of ventricular arrhythmias at MIS-C. Similar to our data, increased QT dispersion, Tp-e interval, and Tp-e/QTc ratio are markers of susceptibility to ventricular arrhythmias and have been identified in previous studies [10-14,21,22].

One of the patients included in the study was found to have severe ventricular tachycardia and ventricular fibrillation. Previous studies have identified cases of ventricular tachycardia with significant hemodynamic effects in patients with MIS [23,24]. Increased variability in ventricular repolarization is associated with increased ventricular arrhythmias. Increased QTc, QT dispersion, Tp-e interval, and Tp-e/QTc ratio in our study may be associated with the risk of ventricular arrhythmias in these patients. In addition, an association was found between increased QT dispersion, Tp-e interval, and Tp-e/QTc ratio, and decreased ejection fraction. The severity of impairment of left ventricular systolic function may contribute to increased ventricular arrhythmia [25]. Similarly, several reports on the cardiovascular involvement of this disease have discussed that MIS-C patients are at an increased risk of severe arrhythmias. It has already been hypothesized that this is likely due to myocardial inflammation and edema, a finding that has been confirmed and characterized by detailed cardiac MRI studies [26].

However, the present study has several limitations. Small sample size, observational study, and low event rate are major limitations of the study. Also, serum levels of NT-proBNP were not measured and MIS-C patients were not monitored for possible ventricular arrhythmias with extended Holter monitoring.

## Conclusions

We demonstrated increased QTc interval, QT dispersion, Tp-e interval, and Tp-e/QTc ratio in MIS-C patients. In addition, a correlation was found between increased QT dispersion, Tp-e interval, and Tp-e/QTc ratio, and decreased ejection fraction. Left ventricular systolic dysfunction may be associated with an increased risk of ventricular arrhythmias. Susceptibility to ventricular arrhythmia is possible at MIS-C. More comprehensive studies are needed to determine this risk.
